# Red Emitting Solid-State CDs/PVP with Hydrophobicity for Latent Fingerprint Detection

**DOI:** 10.3390/ma17081917

**Published:** 2024-04-21

**Authors:** Zhihong Zhang, Zhaoxia Han, Shuhui Ding, Yujing Jing, Zhenjie Wei, Dawei Zhang, Ruijin Hong, Chunxian Tao

**Affiliations:** 1 Shanghai Key Lab of Modern Optical System, University of Shanghai for Science and Technology, Shanghai 200093, China; 2 Engineering Research Center of Optical Instrument and System, Ministry of Education, Shanghai 200093, China

**Keywords:** carbon dots, red fluorescence, hydrophobicity, latent fingerprints

## Abstract

Fluorescent carbon dots (CDs) are a new type of photoluminescent nanomaterial. Solid-state CDs usually undergo fluorescence quenching due to direct π-π* interactions and superabundant energy resonance transfer. Therefore, the preparation of solid-state fluorescent CDs is a challenge, especially the preparation of long wavelength solid-state CDs. In this research, long wavelength emission CDs were successfully synthesized by solvothermal methods, and the prepared CDs showed good hydrophobicity. The composite solid-state CDs/PVP (Polyvinyl pyrrolidone) can emit strong red fluorescence, and the quantum yield (QY) of the CDs/PVP powder reaches 18.9%. The prepared CDs/PVP solid-state powder was successfully applied to latent fingerprint detection. The results indicate that the latent fingerprints developed by CDs/PVP powder have a fine definition and high contrast visualization effect, which proves that the prepared CDs/PVP has great application potential in latent fingerprint detection. This study may provide inspiration and ideas for the design of new hydrophobic CDs.

## 1. Introduction

As a new form of carbon-based nanomaterials, carbon dots (CDs) have been widely used in bioimaging, optoelectronic devices, sensing, photocatalysis and other fields [1]. Due to their stable luminescent performance, abundant surface functional groups, safety and nontoxicity, good biocompatibility and low cost [2], fluorescent CDs have great application prospects in the field of latent fingerprints (LFPs) detection and recognition. Human fingerprints show uniqueness and lifelong invariance, and thus offer one of the most reliable means of personal identification. Therefore, fingerprints play an important role in identity recognition and the investigation of criminals [3]. LFPs are fingerprint marks formed by natural bodily secretions such as sweat and oil remaining on contact materials, which are one of the key evidences used in crime scene investigations. Due to the poor optical contrast between LFPs and the surface of objects, they are difficult to distinguish with the naked eye under visible light. They must be extracted by physical or chemical methods.

Fingerprint detection technology has profound use in forensic science and criminal investigation fields, among which fingerprint collection is the most important step. Traditional LFPs collection techniques including ninhydrin [4], iron oxide [5] and fluorescent dyeing [6] have many shortcomings, such as low contrast, low sensitivity, low selectivity and high toxicity. Some fluorescent dyes have potential toxicity or show fluorescence quenching when aggregated, which limits their use in LFPs detection. Therefore, it is necessary to find fluorescent materials with good biocompatibility and bright solid-state fluorescence for LFPs extraction [7]. In recent years, CDs have been explored and used in the field of LFPs detection because of their excellent optical properties, lower toxicity and better biocompatibility.

However, most CDs that have been reported emit blue or green fluorescence under ultraviolet excitation. Under the irradiation of an ultraviolet lamp, the background usually emits blue or green fluorescence, which will interfere with and reduce the clarity of developing fingerprints. CDs emitting red fluorescence can reduce background interference and significantly enhance the contrast of LFPs. But when the CDs solution is dried, the obtained CDs powder tends to undergo fluorescence quenching due to direct π-π* interactions or excessive resonance energy transfer [7], which is called the aggregation-caused fluorescence quenching (ACQ) effect. Research has shown that the ACQ effect can be effectively overcome by physical or chemical doping. Zhai and his colleagues used montmorillonite (MMT) as the dopant; the CDs were uniformly dispersed in the lamellar structure of the MMT clay matrix, which effectively prevented the ACQ effect [8]. Li and his colleagues doped with starch, and the composites overcame the oxidation tendency of quantum dot powders such as CdTe in the air [9].

At present, there are many methods used for LFPs development, such as the fingerprint powder method, small particle suspension method, transfer method, and spray method [10]. In this research, red-emitting CDs were successfully synthesized using a solvothermal method. In order to overcome the ACQ effect, the CDs solution was mixed with polyvinyl pyrrolidone (PVP) and dried to obtain solid-state CDs/PVP fluorescent powder. The solid-state CDs/PVP powder can emit strong red fluorescence, and was successfully applied in LFPs development and recognition. The results indicate that the CDs/PVP had excellent hydrophobicity, and could maintain long-term stability and availability in a humid environment, proving that the prepared CDs/PVP have great application potential in LFPs detection. This research may also provide inspiration and ideas for the design of new hydrophobic CDs.

## 2. Experimental

### 2.1. Materials

O-Phenylenediamine (OPDA, CP, 98.5%), phthalic acid (AR, 99.8%), ethanol (AR, 99.7%), dimethyl formamide (DMF, AR, 99.5%) and dilute nitric acid (GR, 65%) were purchased from Sinopharm Chemical Reagent Co., Ltd. (Shanghai, China). Polyvinyl pyrrolidone (PVP) with an average molecular weight of 10,000 and specifications of K13-18 was purchased from Aladdin Chemistry Co., Ltd. (Shanghai, China). All chemicals and reagents were used directly without further purification. Deionized water was used throughout the experiment.

### 2.2. Synthesis of Carbon Dots

The CDs were synthesized through a solvothermal method. In a typical experimental procedure, 1.35 g OPDA and 0.415 g Phthalic acid (molar ratio 5:1) were added in 20 mL ethanol, and then stirred and sonicated to obtain a uniform solution. Then, 2 drops of dilute nitric acid were added to the mixed solution; the mixed solution was then transferred into 40 mL Teflon-lined stainless-steel autoclaves and heated at 200 °C for 6 h. After the reaction was completed, the autoclave was cooled down to room temperature and opened. The supernatant and impurities were discarded, and the inside of the reactor was rinsed with deionized water. The black solid attached to the inner wall and bottom of the reactor was collected and dissolved in 50 mL ethanol. Then the ethanol solution was poured into a dialysis bag for dialysis in a big beaker to remove residual organic molecules, and we replaced the deionized water in the beaker after 2, 6, and 12 h. The solution after dialysis was centrifuged at 10,000 rpm for 5 min; the black precipitate at the bottom of the centrifuge tube was dissolved in ethanol. This was the CDs solution, which can emit bright red fluorescence under 365 nm UV (Ultraviolet) light. The CDs solution was then dried in a vacuum oven at 50 °C for 24 h. The obtained CDs powder was stored in a drying cabinet at room temperature for further characterization. In this study, inner wall attachments with better performance were selected and reaction solutions with low emission peaks and low quantum efficiency were discarded.

### 2.3. Preparation of CDs/PVP

In order to overcome the fluorescence quenching of solid-state CDs, physical doping can be applied to the as-synthesized CDs to eliminate the ACQ effect. In the present study, PVP was found to be the most suitable material for doping solid-state fluorescent CDs. The reasons are as follows: (1) With a large chain-like structure, PVP can maintain an appropriate distance between CDs to eliminate solid-state CDs fluorescence quenching. (2) PVP has abundant functional groups, it is easily soluble in ethanol and it has good compatibility with CDs. We added 2 g of PVP to the CDs solution, mixed it well with the ultrasonic method and then dried it. The dried powder was collected and then ground in an agate mortar to obtain CDs/PVP powder. The fine ground CDs/PVP powder could overcome fluorescence quenching because the CDs can be evenly dispersed in PVP to avoid aggregation.

**Figure 1:**
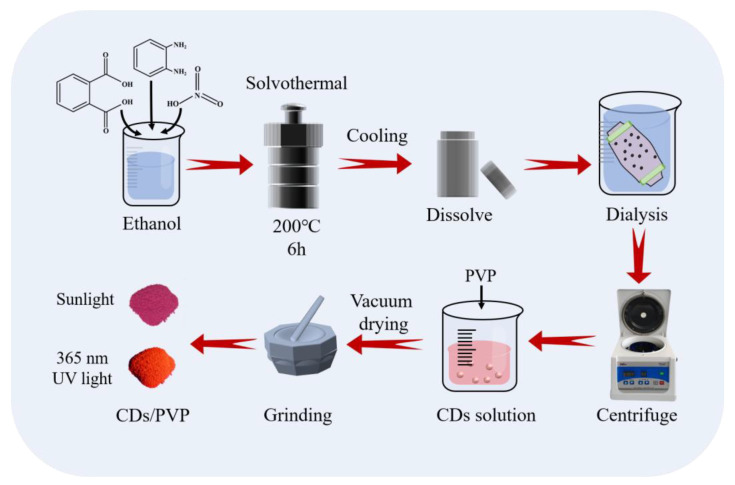
The diagram of the preparation for CDs and CDs/PVP.

The diagram of the mode of preparing CDs and CDs/PVP is shown in Figure 1. Photos of the CDs/PVP powder under sunlight and 365 nm UV light are also shown. The prepared CDs/PVP powder showed a good photoluminescence performance, and could emit strong red fluorescence, enabling it to overcome the influence of traditional substrate background fluorescence and generate clear LFPs images.

**Figure 2:**
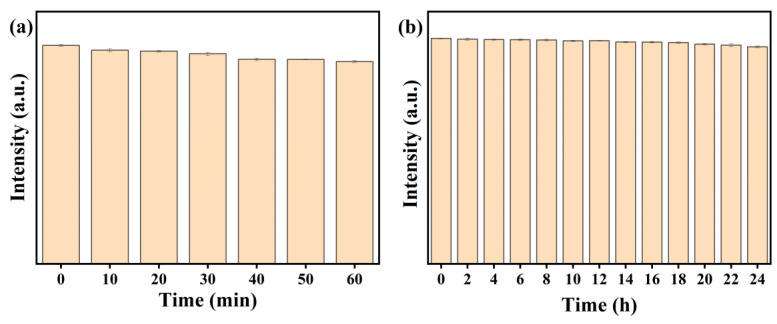
The fluorescence intensity of CDs/PVP under 365 nm UV light for 60 min (**a**) and sunlight for 24 h (**b**).

The prepared CDs/PVP shows good fluorescence stability. As shown in Figure 2, it can be seen that the fluorescence intensity of the CDs/PVP decays very slightly after irradiation for 60 min under UV light and 24 h under sunlight. After undergoing the irradiation experiments, the luminescence intensity remained stable over a long period of time, indicating that CDs/PVP is suitable for use in the LFP application.

### 2.4. Visualization of Latent Fingerprints

To prepare LFPs, volunteers first cleaned their hands with soap and let them dry, then rubbed their fingers on the greasy areas of their faces. Next, they pressed their fingers on the substrates with appropriate pressure to obtain LFPs. The LFPs were visualized using the classic powder spreading method. Firstly, CDs/PVP powder was gently sprinkled on the LFPs. After a few seconds, the excess powder was carefully removed with a fingerprint brush. Under UV light irradiation, bright red fluorescent fingerprint patterns could be clearly seen.

### 2.5. Characterization

Transmission electron microscopy (TEM, JEM-3200FS, Japan Electronics, Tokyo, Japan) was used to study the morphology of CDs. The X-ray diffraction pattern of CDs was recorded with a Cu Kα radiation X-ray diffractometer (XRD, MiniFlex600, Rigaku, Tokyo, Japan). The absorption spectrum was measured using a UV-Vis (Ultraviolet-Visible) spectrophotometer (Lambda1050, Perkin-Elmer, Waltham, MA, USA). Quantum yield measurements were performed on a fluorescence spectrometer (Dual UV-NIR, Horiba, Piscataway, NJ, USA) with a calibrated integrating sphere. The X-ray photoelectron spectrum was measured using X-ray photoelectron spectrometer (XPS, K-Alpha, Thermo Fisher Scientific, Waltham, MA, USA). The Fourier transform infrared spectrum was measured using fourier transform infrared spectrometer (FTIR, IS5, Thermo Fisher Scientific, Waltham, MA, USA). The fluorescence decay curve of CDs was measured using an steady state/transient fluorescence spectrometer (FLS1000, Edinburgh Instruments, Livingston, Scotland, UK) steady-state/transient fluorescence spectrometer with a xenon lamp as the excitation source, and its fluorescence lifetime was calculated.

## 3. Results and Discussion

### 3.1. Effects of Molar Ratio and Concentration on CDs Synthesis

As shown in Figure 1, CDs were synthesized by the solvothermal method using OPDA, phthalic acid and nitric acid as raw materials. In order to investigate the effects of molar ratio and concentration on CDs synthesis, a series of experiments were designed and carried out under different reaction conditions. The experimental parameters for CDs synthesis and the optimal emission wavelength are shown in Table 1. When the molar ratio of OPDA to phthalic acid was 2:1, there was no black reaction precipitate, and the optimal emission wavelength of the reaction solution was 569 nm. A small number of CDs adhered to the inner lining of the reaction vessel when the molar ratio was 3:1 and 4:1, and the optimal emission wavelengths of their ethanol solutions were 597 nm and 569 nm, respectively. However, when the molar ratio was 5:1, a large number of CDs stuck to the inner wall. It was found that the reagents concentration also has a great effect on CDs synthesis. When the reagents concentration was low (OPDA: phthalic acid = 0.45:0.14 g), the optimal emission wavelength of the ethanol solution of CDs that were stuck to the inner wall was 569 nm. As the reagents concentration increased (OPDA: phthalic acid = 1.35:0.415 g or 2.16:0.83 g), the optimal emission wavelengths of the ethanol solution of CDs sticking to the inner wall were both 649 nm. As the molar ratio of the reagents increased, OPDA was in excess with respect to phthalic acid, and thus remained as an impurity in the CDs product. Therefore, we selected the optimized reagent ratio of OPDA to phthalic of 1.35:0.415 g.

We speculate that the possible reaction mechanism is as follows. OPDA and phthalic acid are polymerized through a coupling reaction between -NH_2_ and -COOH, and carbonized through dehydration or deamination to form CDs with a larger conjugated structure. Nitric acid has a strong oxidation ability, which can increase the surface oxidation degree of CDs, thereby transforming the emission wavelength of CDs. The prepared CDs solution emits bright red fluorescence under 365 nm UV light.

### 3.2. Morphology and Microstructure Analysis

**Figure 3:**
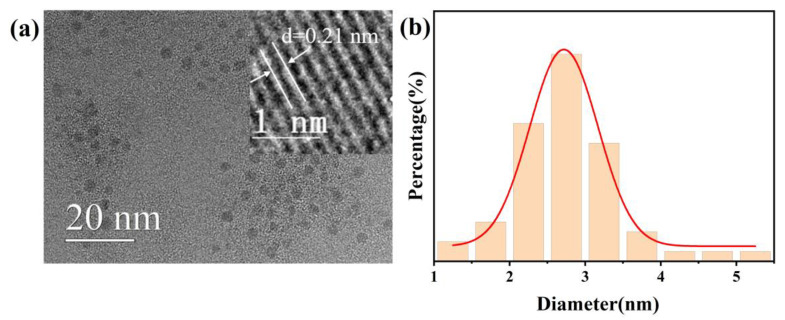
(**a**) TEM and HRTEM images of the CDs; (**b**) particle size distribution diagram and its fitting line of the CDs.

The morphology and particle size distribution of the as-prepared red CDs were observed by TEM. Figure 3a shows the TEM image of the CDs; we can see that the CDs are spherical and uniformly dispersed without obvious agglomeration. The upper right inset in Figure 3a shows the corresponding high-resolution TEM (HRTEM) image, which has clear lattice fringes. The fringe spacings of 0.21 nm closely match the (100) lattice distance of graphitic carbon [10]. Figure 3b shows a statistical graph of particle size; the average size of the CDs is 2.75 nm.

**Figure 4:**
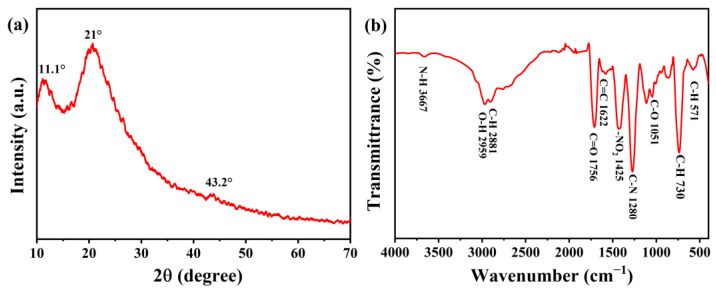
(**a**) XRD pattern of the CDs; (**b**) FTIR spectrum of the CDs.

The structure of the as-synthesized CDs was investigated by XRD. Figure 4a shows the XRD pattern of the CDs; there is a weak diffraction peak at 11.1° and a strong diffraction peak at 21°. The diffraction peak at 11.1° is the (001) characteristic peak of graphite oxide, indicating that the graphite was transformed into graphite oxide through the oxidation reaction [11]. The diffraction peak position at 21° indicates the sp^3^ in the graphene plane, which improves the interlayer distance [12]. There is a micro-peak at 43.2°, which belongs to the carbon (100) plane [13].

In order to further analyze the internal structure and surface functional groups of the CDs, the CDs powders were characterized by FTIR. Figure 4b shows the FTIR spectrum; the absorption peak at ~3667 cm^−1^ belongs to the amino characteristic absorption peak, which can be attributed to N–H stretching vibration [14,15]. The absorption peak at ~2959 cm^−1^ is attributed to the stretching vibration of O–H. The two absorption peaks at ~1756 cm^−1^ and ~1622 cm^−1^ are attributed to the stretching vibration of C=O and the backbone vibration of aromatic C=C, respectively, indicating the carbonyl functional group [16]. In addition, the absorption peak at ~1280 cm^−1^ is attributed to the stretching vibration of C–N. The absorption peak at ~1425 cm^−1^ is attributed to the -NO_2_ nitro compound. The absorption peak at ~1051 cm^−1^ is attributed to the C–O vibration. The absorption peaks at ~730 cm^−1^ and ~571 cm^−1^ are attributed to the deformation vibrations of the C–H aromatic ring [17].

**Figure 5:**
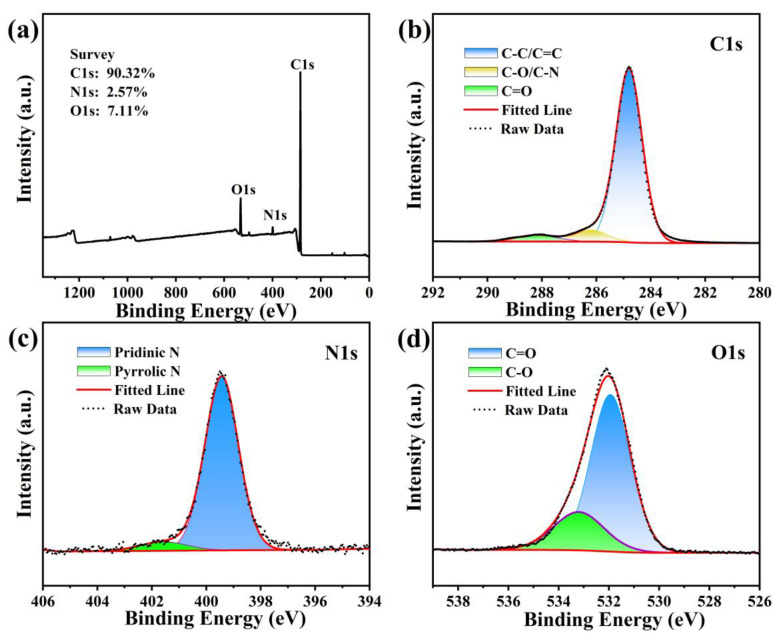
(**a**) XPS survey spectrum of the CDs; (**b**) C1s high-resolution XPS spectrum; (**c**) N1s high-resolution XPS spectrum; (**d**) O1s high-resolution XPS spectrum.

To explore the element composition, chemical bonds and functional group properties on the surfaces of the CDs, the CDs were examined by XPS. Figure 5a shows the XPS survey spectrum; the three main peaks are located at 284.8 eV (C1s), 399.4 eV (N1s) and 532.4 eV (O1s), which signifies that the primary components of the CDs are C (90.32%), N (2.57%) and O (7.11%). We can see that the contents of C and O elements in CDs have increased compared to the raw materials, which may be due to the coupling reaction of functional groups during the CDs formation process. Respectively, Figure 5b–d exhibit the element C1s, N1s, O1s XPS high-resolution spectra of the CDs. The element C1s spectrum can be decomposed into three peaks according to the peak fitting. The peak at 284.8 eV is classified as sp^2^ C (C-C/C = C), which indicates that there are large numbers of stratiform graphite structures at the core of the CDs. Located at 286.2 eV, this peak is formed from sp^3^ C (C-O/C-N). The last peak at 288.2 eV is derived from C=O bonds, indicating a lot of carbon-containing and oxygen-containing functional groups attached to the surfaces of the CDs. The N1s element spectrum is made up of two peaks related to the peak fitting. One peak is at 399.4 eV, and another is at 401.6 eV. They can be related to pyridinic N and pyrrolic N, showing that the CDs successfully fix and couple the N element [18,19]. In the O1s spectrum, two peaks emerge that are based on peak fitting. The C=O bonds are indicated by the peak position of 531.3 eV and the C–O bonds by the peak position of 532.4 eV, implying the presence of hydroxyl and carboxyl groups. The presence of C–O, C–N and C=C functional groups causes the CDs to have a certain degree of hydrophobicity [20]. The XPS results are consistent with the findings of the FTIR analysis—the CDs’ surfaces feature rich functional groups containing oxygen and nitrogen.

### 3.3. Optical Property Studies

**Figure 6:**
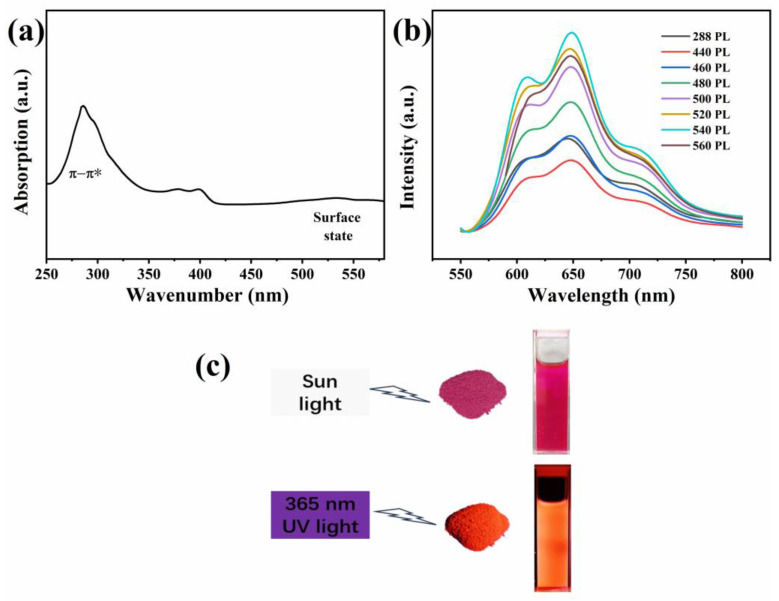
(**a**) UV-Vis absorption spectrum of the CDs. (**b**) The emission spectra of the CDs/PVP at different excitation wavelengths. (**c**) CDs/PVP powder and CDs ethanol solution photos under sunlight and 365 nm UV light.

The UV-Vis absorption spectrum of the CDs is shown in Figure 6a. The absorption peak at 288 nm is derived from the π-π* electronic transition of the aromatic sp^2^ structural domains (C–C), with the absorption peak at 540 nm assigned to increased n-π* transitions in the C=O- and C=N-related surface state [21,22]. Figure 6b shows the fluorescence emission spectra of the CDs/PVP at different excitation wavelength. Figure 6b shows that the fluorescence emission intensity of the CDs/PVP first increases and then decreases as the excitation wavelength increases. When the excitation wavelength is 540 nm, the fluorescence emission intensity reaches its maximum. The strongest emission peak is at 649 nm, indicating that the CDs/PVP emits red fluorescence. Combined with Figure 6a,b, it can be seen that strong absorption at 288 nm corresponds to a weak fluorescence emission, which indicates that the CDs undergo π-π* direct interaction or excessive resonance energy transfer from the liquid to the solid state, resulting in a sudden decrease in the emission intensity [23]. In addition, the CDs/PVP exhibits excitation-independent photoluminescence characteristics. Figure 6c shows photos of the ethanol solution of the CDs and CDs/PVP powders under sunlight and 365 nm UV light. It can be seen that they are pink in sunlight, and they are red under 365 nm UV light. Related studies have shown that the formation of phenyl red fluorescence CDs depends on the molecular mechanism. During high-temperature synthesis, the phenyl precursor forms a molecular fluorophore, which has the same luminescent properties and behavior as red fluorescent CDs [24,25].

**Figure 7:**
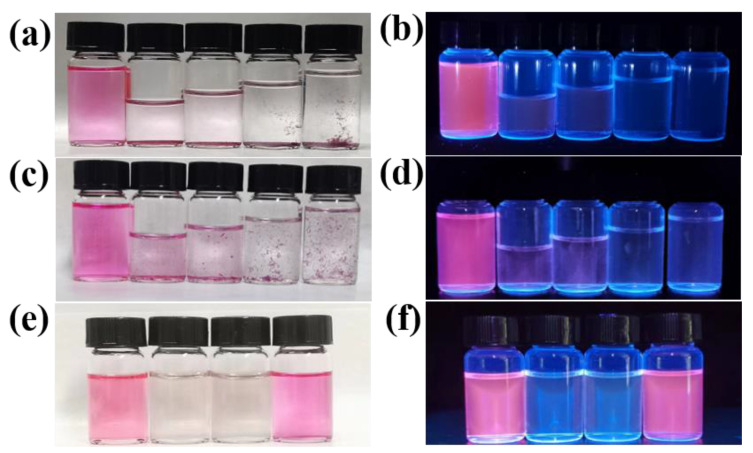
Photos of CDs solutions with different volume ratios of water to ethanol (0, 3:1, 5:1, 7:1 and 9:1, from left to right) under sunlight (**a**) and UV light (**b**). After shaking uniformly, the photos of the CDs solution under sunlight (**c**) and UV light (**d**). Photos of the CDs DMF solution, CDs water solution, CDs NaCl (1 mol/L) solution, and CDs ethanol solution under sunlight (**e**) and UV light (**f**).

The CDs’ aggregation phenomenon was discovered in a special solution with a high water ratio, indicating its hydrophobicity. The hydrophobicity experiment was conducted as follows. Firstly, five samples of 0.4 mL of CDs ethanolic solution were transferred into five glass vials. Then, increasing volumes of 0 mL, 1.2 mL, 2 mL, 2.8 mL and 3.6 mL water were added to the vails from left to right, and mixed well. Finally, 3.6 mL of ethanol was added to the leftmost bottle to serve as a control. Figure 7a,b show photos of the CDs solutions with different water ratios under sunlight and UV light. respectively. It can be seen that there are precipitates on the bottoms of the glass bottles containing water. After shaking uniformly, photos were taken, as shown in Figure 7c,d. We can see that the fluorescence of the CDs solution gradually changes from red to light pink and then to transparent with the increase in water. Meanwhile, the CDs are uniformly dispersed in the ethanol organic solvent, but not in water. Figure 7e,f show photos of the CDs DMF solution, CDs water solution, CDs NaCl (1 mol/L) solution, and CDs ethanol solution under sunlight and UV light, respectively. The aggregation state can be observed by the naked eye, verifying the hydrophobicity of the CDs and their different solubilities in organic solutions [26].

**Figure 8:**
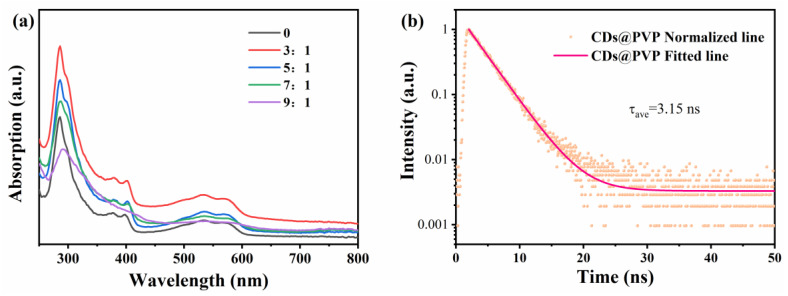
(**a**) The absorption spectra of the CDs solutions with different volume ratios of water to ethanol. (**b**) The fluorescence lifetime decay curve of CDs/PVP.

Figure 8a gives the absorption spectra of the CDs solution with different water ratios. The absorption peaks of the CDs ethanol solution appear at 288 nm, 402 nm and 540 nm. As the water ratios increase, the absorption peak at 288 nm gradually redshifts to 298 nm, and the absorption peak at 540 nm gradually redshifts to 556 nm. Meanwhile, the absorption peak flattens out at 402 nm, indicating that the absorption peaks are related to the aggregation of CDs. The formation of CD aggregates in the solution with a high water fraction confirms its hydrophobicity, caused by the restriction of the intramolecular rotation and activation of AIE (Aggregation Induced Emission) [27]. Further, the photoluminescence quantum yield (QY) of CDs/PVP was measured using the absolute method. At the optimal excitation of 540 nm, the measured absolute QY of CDs/PVP was 18.9%. Fluorescence decay is one of the inherent properties of the emission state. Therefore, the fluorescence lifetime decay curve of the CDs/PVP was also investigated, as shown in Figure 8b. It can be seen that the fluorescence lifetime of CDs/PVP decays biexponentially, and the average fluorescence lifetime $\tau_{ave}$ was 3.15 ns. The solid line in Figure 8b is a second-order function fitting curve, which is fitted according to the following equation [28]:

$$y=y_{0}+A_{1}e^{-\left(x-x_{0}\right)/\tau_{1}}+A_{2}e^{-\left(x-x_{0}\right)/\tau_{2}}$$

where *y* is the fluorescence emission intensity of CDs/PVP at moment t, *y*_0_ is a constant, *A*_1_, *A*_2_, *x*_0_ are the coefficients, and *e* is the natural constant. $\tau_{1}$ and $\tau_{2}$ are the fluorescence emission lifetimes of the carbon nucleus eigenstate and surface functional group of CDs/PVP, respectively.

### 3.4. Application in Latent Fingerprint Detection

**Figure 9:**
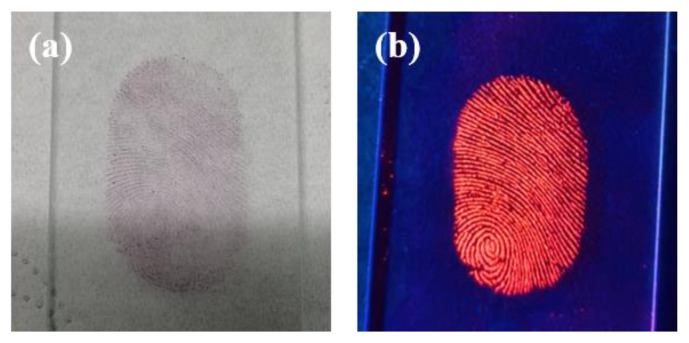
(**a**) LFPs image on glass under sunlight; (**b**) LFPs image on glass under 365 nm UV light.

Based on the excellent luminescent properties of CDs/PVP, LFPs were developed using a powder sprinkling and sweeping technique. Firstly, we prepared LFPs of volunteers according to the method described in Section 2.4. Then, CDs/PVP powder was evenly sprinkled on the LFPs, and excess CDs/PVP powder was carefully removed with a fingerprint brush. Various substrates, such as paper money, coins, tinfoil paper, rulers, glass and plastic were tested, and a very clear LFPs image was obtained on the glass. Figure 9 shows images of the development of LFPs on the glass under sunlight and 365 nm UV light. The LFPs with CDs/PVP emitted intense red fluorescence under 365 nm UV light.

In this research, LFPs are identified using a three-level classification approach. As shown in Figure 9a, we can determine the locations of fingerprint cores and scars using the image of LFPs under sunlight. Due to the powder clustering phenomenon, it is difficult to determine detailed fingerprint features. Therefore, relying on these signs alone is not yet sufficient to discriminate between individual human beings. Figure 9b shows an LFP image under 365 nm UV light; it can be seen that there is a high contrast between the fingerprint lines and the glass substrate, which enables detailed feature information such as bifurcation, islands and ridge ends to be easily identified with the naked eye, and the observer is able to make preliminary judgments on ridge details including the ridge distances and their relative positions.

**Figure 10:**
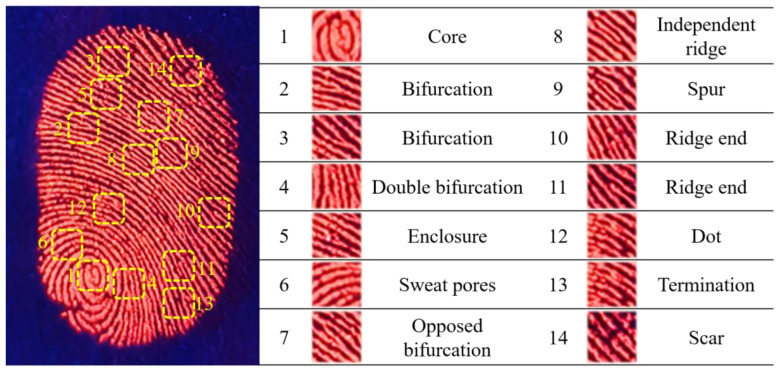
Enlarged fluorescence image and details of latent fingerprint on glass substrate.

Figure 10 shows an enlarged fluorescence image and details of the LFP on the glass substrate, which was analyzed in detail to identify the first-, second- and third-level features. The red fluorescence emission greatly enhances the contrast between the fingerprint lines and the glass substrate, providing a significant visual difference. The core pattern and bucket pattern of fingerprint shape can be identified via the first-level features. In Figure 10, the core pattern can be clearly seen. The second-level features, such as bifurcation, double bifurcation, enclosure, opposed bifurcation, independent ridge, spur, ridge end, dot and termination, are visible to the naked eye in the magnified images on the right. The third-level features, such as sweat holes and scars, are also clearly visible. The results show that the LFPs on glass substrate are easy to identify and have high imaging quality. CDs/PVP powder firmly adheres to the various fingerprint lines due to electrostatic interactions. A great many functional groups exist on the surfaces of CDs/PVP, including amino, carboxyl and hydroxyl groups. Sweat pores on the surfaces of the LFPs prove the presence of a water–oil mixture and proteins [29]. As a result, the hydrophobic carbon dots bind well to fingerprinted grease. The electrostatic interactions between the water–oil mixture, proteins and CDs/PVP powder enable LFP development, thus providing a convenient way to extract individual features.

**Figure 11:**
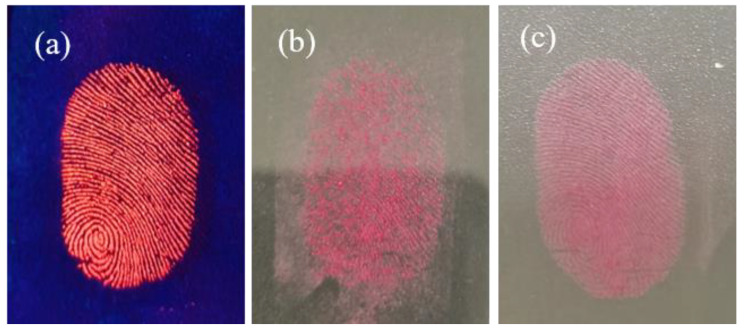
Fingerprint images developed using CDs/PVP (**a**) under 365 nm UV light after 10 days, (**b**) under sunlight after 3 months in a humid environment, and (**c**) under sunlight after re-drying.

The LFP images taken under different conditions and developed using CDs/PVP are shown in Figure 11. As depicted in Figure 11a, the great fluorescence performance of the LFP was maintained after 10 days. In Figure 11b, the obvious agglomeration of CDs/PVP can be seen on LFPs stored in humid conditions after three months. However, after simple heating and drying, the moisture content of CDs/PVP powder is greatly reduced, and clear LFPs can be successfully collected, as shown in Figure 11c. The LFP retains clear fluorescence emission under UV irradiation. It can be concluded that the hydrophobicity of CDs is the main factor allowing them to overcome the defect of deliquescence in humid environments.

**Figure 12:**
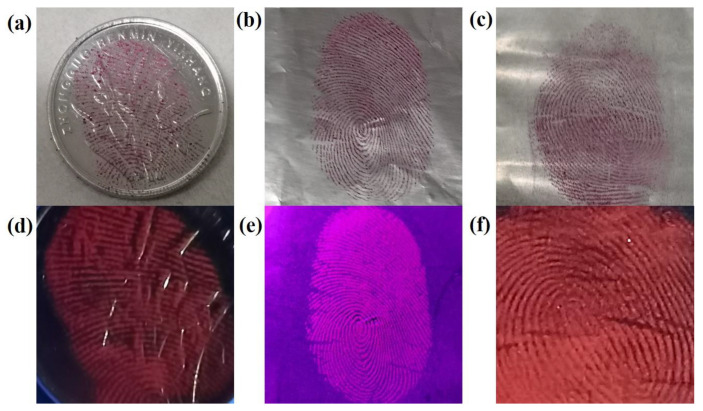
LFP images developed using CDs/PVP on an engraved coin, tinfoil and plastic film under sunlight (**a**–**c**) and 365 nm UV light (**d**–**f**).

Focusing on the complexity of crime environments, we also tested more application scenarios. Figure 12 shows the LFP images developed using CDs/PVP on engraved coins, tinfoil, and plastic film under sunlight (a–c) and 365 nm UV light (d–f). It can be seen that these LFP images are relatively clear, and details such as the shape of the core and the locations of scars and bifurcations are easily identified. In particular, a coin with a carved pattern on its surface can offer a clear fingerprint image.

## 4. Conclusions

Using the one-step solvothermal method, red light-emitting fluorescent CDs were synthesized in ethanol using OPDA and phthalic acid as the reagents. The synthesized CDs were spherical in shape, and their average size was 2.75 nm. Then, using long-chain PVP as the doping material, a CDs/PVP solid-state fluorescence powder was successfully prepared, which successfully resolved the ACQ effect arising after PVP doping, and can emit bright red fluorescence at 649 nm under 540 nm light irradiation. The fluorescence quantum yield of the solid-state CDs/PVP powder was 18.9%. Due to its excellent hydrophobic property, the CDs/PVP can maintain long-term stability and availability in an air environment. The prepared CDs/PVP has been successfully used for rapid LFPs detection. The results show that the LFPs developed using CDs/PVP powder have high clarity and contrast, and can be used to effectively identify three levels of LFP details. The prepared CDs/PVP shows great potential and advantages related to its use in practical fingerprint detection applications in the future.

## Figures and Tables

**Table 1:** Experimental parameters set for CDs synthesis.

Mole Ratio of Reagents (OPDA:Phthalic Acid)	Mass Ratio of Reagents (OPDA:Phthalic Acid)	Reaction Conditions	Optimal Emission Wavelength (nm)
2:1	0.504:0.415 g	200 °C, 6 h	569
3:1	0.81:0.415 g	200 °C, 6 h	597
4:1	1.08:0.415 g	200 °C, 6 h	569
5:1	0.45:0.14 g	200 °C, 6 h	569
5:1	1.35:0.415 g	200 °C, 6 h	649
5:1	2.16:0.83 g	200 °C, 6 h	649

## Data Availability

The data of this paper are available on request from the corresponding author.
